# Socioeconomic differentials and mortality from colorectal cancer in large cities in Brazil

**DOI:** 10.3332/ecancer.2016.614

**Published:** 2016-01-20

**Authors:** Viviane Gomes Parreira, Karina Cardoso Meira, Raphael Mendonça Guimarães

**Affiliations:** 1 Fundação Oswaldo Cruz, Instituto Nacional de Infectologia Evandro Chagas, Brazil; 2 Universidade Federal do Rio Grande do Norte, Escola de Enfermagem de Natal, Brazil; 3 Fundação Oswaldo Cruz, Escola Politécnica de Saúde Joaquim Venâncio, Brazil

**Keywords:** mortality, colorectal cancer, social indicators

## Abstract

The objective of this study was to compare the mortality pattern of colorectal cancer according to the social development profile of the large Brazilian cities. This was an ecological study that used as units of analysis Brazilian municipalities that were considered to be large (i.e. over 100,000 inhabitants). The social indicators adopted were obtained from the Atlas of Human Development in Brazil. Mortality data came from the Mortality Information System (MIS), represented by codes C18, C19, and C20. For data analysis, municipalities were characterised according to the indicator profile used by multivariate classification cluster analysis. It was observed that the Southeast, South, and Midwest regions concentrated over 90% of cities in the group of more developed municipalities, while the North and Northeast regions were represented by 60% of cities in the group of less developed municipalities. The mortality pattern of colorectal cancer in both groups was different, with a higher average mortality rate from colorectal cancer for populations living in cities from the more developed group (*p* = 0.02). The mortality rate from this cancer was shown to be directly proportional to the Municipal Human Developlemnt Index (MHDI) and inversely proportional to the inequality indicator (*p* < 0.001); therefore the highest means were observed among the municipalities with better socioeconomic conditions. It is important to consider social disparities to ensure equity in healthcare policy management.

## Background

The relationship between the different socioeconomic levels and the occurrence of cancer is a result of the interaction between various issues [1, 2, 5]. In particular, the cultural patterns among social classes influence this relationship. Lifestyle and exposure to cancer risk factors have been changing rapidly in recent years. These alterations are uneven among different areas, reinforcing regional differences, mainly in metropolitan areas [1].

In relation to the morbimortality profile of cancer, social inequalities are most striking in the strata with the worst socioeconomic indicators. It is in the lower social strata that the difficulties of access to healthcare services for diagnosis, therapy, and rehabilitation become more evident [2].

Colorectal cancer is the third most common cancer worldwide, accounting for approximately 1.4 million new cases in 2012, of which 746,000 were diagnosed in men and 614,000 in women [3].

Regarding the incidence of cancer in Brazil, the National Cancer Institute (INCA) estimates that in 2015, 15,070 men and 17,530 women will have colorectal cancer, representing the third and second anatomic locations respectively. Among Brazilian regions, the Southeast had the highest standardised incidence rate of colorectal cancer for males (22.67/100,000) and females (24.56/100,000). Smaller standardised incidence rates were observed in the North: 4.48/100,000 and 5.30/100,000, respectively, among men and women [4].

In Brazil, mortality from colorectal cancer is distributed unevenly among the states and capitals, and depending on the geographic region, resembles both developed and developing countries. Several studies have demonstrated a steady increase in mortality rates for this type of cancer in Brazilian regions, especially in the South and Southeast, which presented rates similar to those of highly industrialised countries [5, 6]. Socioeconomic standards of individuals or population groups are evaluated through indicators, as they allow for international comparisons. Indicators can be analytical, when they are made up of a single variable (life expectancy at birth, literacy rate, mean education), or synthetic, when they are a result of a composition of variables, such as the Human Development Index (HDI), which is based on income, occupation, education, and longevity data [7].

Differences in patterns of cancer incidence and mortality are observed between areas with higher and lower social development worldwide [8]. The process of economic development stimulates advances in medical technology and improved access to health care, both for infectious and non-communicable diseases such as cancer [9]. However, few comparative studies have been conducted recently on the mortality rates of colorectal cancer in more and less developed areas. Therefore, this study is aimed to compare the mortality pattern of this type of cancer and its correlation according to the social development profile of the large Brazilian cities.

## Methods

This was an ecological study that used as units of analysis Brazilian municipalities considered to be large, i.e., with over 100,000 inhabitants according to the criteria of the Brazilian Institute of Geography and Statistics (IBGE) [10]. A total of 287 municipalities were included in the analysis, distributed in the five Brazilian macro-regions. Considering that the study aimed to observe indicators that were a proxy of the contextual effect of the social situation, the authors chose to assess municipal aggregates. Therefore the analyses can be extrapolated only for the aggregate level and not for the individual level, thereby avoiding the ecological fallacy in the discussion.

The social indicators adopted in the present study were obtained from the Atlas of Human Development in Brazil. Among the indicators of the Atlas, this study adopted the MHDI, which is a summary measure of long-term progress calculated by the geometric mean of three dimensions related to longevity, education, and income. To analyse the development of a country or even a municipality, the HDI takes into account more than just economic factors. It considers life expectancy as a proxy measure of a long and healthy life (health) which inturn is derived by the standard of living (income) as measured by Gross National Income (GNI) per capita expressed in purchasing power parity (PPP) in US dollars, using 2005 as the reference year, and the access to knowledge (education) as measured by: i) mean years of adult education, which is the mean number of years of education of people aged 25 years and older; and ii) the expected years of schooling for children at the age of starting school life [11, 12].

The study also included the Gini coefficient (used to measure the inequality of the distribution of household income per capita. Its value ranges from 0 when there is no inequality to 1 when there is maximum inequality), the urbanisation level (the percentage of population living in urban areas, in a particular geographical space), and the income ratio (expresses the concentration of personal income, by comparing the extreme income strata, that is the higher the value, the greater the income gap among population groups in the considered strata).

The indicators of this study were chosen based on their coverage, according to the literature used, as they encompass information related to health, education, and the phenomena that make up an economic system. All social indicators used are based on the 2010 census.

Unlike the synthetic social indicators, mortality indicators were calculated based on micro-data from the Mortality Information System through the Tabnet application of the Brazilian Ministry of Health, classified in accordance with the tenth revision of the International Statistical Classification of Diseases and Related Health Problems (WHO, 1995). To calculate the rates, the present study included deaths whose records stated malignant colorectal neoplasm as underlying cause, and they were represented by the codes C18, C19, and C20 (including all subcategories) from 2010–2013.

The annual demographic information used, according to age and municipality of residence, were obtained by an inter-census projection made by the IBGE. The cumulative incidence rate for deaths during the study period was calculated.

The raw and standardised coefficients of mortality per 100,000 inhabitants were also calculated. For comparison purposes, the mortality rates were standardised using the direct method; the world population proposed by Segi [13] and modified by Doll *et al* [14] was adopted as standard.

For the analysis, the municipalities that make up the set of locations studied were characterised, according to the profile of the indicators used, by multivariate classification cluster analysis through the K-means method [15] in the statistical package SPSS, version 21. Thus two distinct groups were formed outlining different development profiles.

Multivariate classification, also called cluster analysis, aimed to identify relatively homogeneous groups of cases (in this study, municipalities) based on selected characteristics (in this study, socioeconomic variables). These clusters were defined according to criteria based on distance. This study adopted the non-hierarchical K-means method, which uses the Euclidean distance to define the centre of the groups.

Using the global mean of the indicators (HDI, Gini coefficient, degree of urbanisation, and income ratio) as a reference, the clusters were classified as high or low, and were then classified according to their characterisation regarding the indicators that outlined a social context (good or bad).

From the classification of clusters, average mortality rates adjusted for colorectal cancer clusters were compared having two one-sided hypotheses:

$$\begin{array}{c} H_{0}:\;{\overset{─}{X}}_{m1}=\mu_{m}\;\;\;\;\;H_{0}:\;{\overset{─}{X}}_{m2}=\mu_{m}\\ H_{1}:\;{\overset{─}{X}}_{m1}>\mu_{m}\;\;\;\;\;H_{1}:\;{\overset{─}{X}}_{m2}<\mu_{m} \end{array}$$

Where:

${\overset{─}{X}}_{m1}$ = mean mortality rate of colorectal cancer in cluster 1

${\overset{─}{X}}_{m2}$ = mean mortality rate of colorectal cancer in cluster 2

$\mu_{m}$ = mean mortality rate of colorectal cancer in all municipalities

To test the hypotheses, the Student’s t-test was used and its F-statistic was assessed at a significance level of 5%. To analyse the variability of indicators in more detail, the bivariate Pearson correlation was calculated in order to explain the relationships that are softened in the comparison of means between groups of municipalities.

## Results

Analysis of the grouping of cities by surveying the economic indicators by region verified that the Southeast, South, and West Central regions contained more than 80% of the cities in Group A, while the North and Northeast were represented by more than 90% of the cities in Group B (Table 1).

The socioeconomic indicators of each group of cities can be seen in Table 2.

Of the 287 cities, 235 (82.0%) are concentrated in Group A. This group is the one that showed a population with greater human development (HDI = 0.801) and a lower Gini coefficient, characterising less social inequality. As for the income ratio goes, less inequality in income was observed between population groups as well as a higher level of urbanisation when compared with the cities in Group B. The Southeast region of Brazil contained the majority of the cities (59.5%) in Group A. In contrast, Group B showed less human development (HDI = 0.70), greater social inequality, and greater inequality in income between the population groups, along with a lower level of urbanisation. In contrast to the previous group, most of the cities were located in the Northeast region (78.8%).

According to Table 3, the pattern of mortality because of colorectal cancer in the two groups was different, showing a higher median mortality rate for the populations of residents of the cities which make up Group A.

Finally, in Table 4 analysis of the correlation between the socioeconomic indicators and the mortality rate because of colorectal cancer in the large Brazilian cities studied verified the fact that the mortality indicator correlated with the social indicators selected for the purpose of grouping the cities into clusters. The mortality rate because of this neoplasia was shown to be directly proportional to the value of the MHDI and the level of urbanisation, and inversely proportional to the inequality indicator and the income ratio (*p* < 0.001)

## Discussion

This study found a relationship between socioeconomic conditions and the mortality rate because of colorectal cancer, demonstrating that the populations with higher social and economic development tend to have greater contact with risk factors such as unhealthy lifestyles, inadequate nutrition, smoking, sedentarism, as well as exposure to environmental and individual risk factors, and population ageing [16].

Several authors have already demonstrated the correlation between social determinants such as education, occupation, and income and the risk factors and prevalence of non-transmissible chronic illnesses [17, 18]. In Brazil, the processes of demographic, epidemiological, nutritional transition, urbanisation, and economic and social growth contribute to a greater risk of developing colorectal cancer [19].

Various studies exploring the context of development to explain the dynamic of the incidence and mortality because of specific types of cancers have demonstrated the fact that the pattern of morbidity and mortality because of this group of causes is directly related to the socioeconomic context. Furthermore, some authors have predicted future scenarios for the different levels of socioeconomic development, measured using the HDI in which they demonstrated that the greatest incidence of cancer would be in areas with a very high HDI, which represented nearly 40% of the total global incidence, even though these areas cover only 15% of the global population [20, 21].

The mortality rate because of colorectal cancer was higher in the cities in Group A, in which most of these cities were located in the South, Southeast, and West Central regions. It is worth noting that these are the areas which have a concentration of health services, including those that make up the network of specialty centres; in addition to having a greater life expectancy at birth, strengthening the relationship between cancer and population ageing [22, 23].

In Brazil, Guimarães and his collaborators in a study to estimate the correlation between median per capita income and mortality rate because of colorectal cancer during the period from 2001–2009 observed that there is a statistically significant positive correlation between the more developed regions and the mortality rates because of colon cancer for both sexes [24].

Data from the Family Budget Survey 2002–2003 indicated relevant regional differences in relation to the dietary patterns of Brazilians.The Southern region showed the lowest percentage in the country of the consumption of cereals, legumes, and oils (4.6%), while the highest percentages occurred in the Northeast (9.4%) and West Central (5.9%) regions. The Southern region had the highest percentage of the consumption of industrialised foods (2.3%), equivalent to double the rate found in the Northeast region (1.1%) [22]. Between 2002–2003 and 2008–2009, the percentage of family budgets spent on meat products increased from 18.3–21.9% of the total amount spent on food for the household, while the amount spent on cereals, legumes, and oils decreased from 10.4–8.0%. The comparative data from these two Family Budget Surveys (2002/03 and 2008/09) on the availability of foods for households showed that the differences in dietary patterns remained between the regions, confirming the inequalities in the consumption profiles of Brazilian families [25, 26].

In October of 2015, scientists from ten countries met at the International Agency for Research on Cancer (IARC), and reached conclusions, following the analysis of more than 800 studies published throughout the world, on the carcinogenicity of the consumption of red meats and processed meats [27]. The proportion of the population which consumes red meats varies throughout the world, from less than 5% up to 100%, and this magnitude principally varies according to two factors: the local culture and the purchasing power of the population, which permits access to these types of products, which in general are more expensive than other dietary items such as grains and legumes [28]. These characteristics of the consumption patterns must be considered in the evaluation of the differences found in the mortality rates because of colorectal cancer among the various regions of Brazil, as the South and Southeast regions probably represent lifestyles which can lead to a higher risk of the development of this neoplasia in comparison to other regions, such as the low consumption of fibers and high level of lipids in the diet, a higher consumption of alcohol and higher prevalences of smoking.

The results of the Family Budget Survey of 2008–2009 found that educational level also affected the consumption patterns of Brazilian families. It is observed that the more years of education the person of reference in the family had, the greater the average monthly expense on food. For example, in families in which the person of reference had less than one year of study, the average monthly expenditure was R$ 1,403.42, and for the families in which the person of reference had 11 or more years of study, this total was nearly 207% greater (R$ 4,314.92) [28].

Several epidemiological studies have demonstrated that prevention programmes have been shown to be effective in the reduction of the mortality and incidence of colorectal cancer, but the Ministry of Health still does not consider the establishment of population programmes to prevent this neoplasia to be viable and cost-effective at this time [29]. Late diagnosis is still common and may be related to the access of the population to health services and programmes, and the difficulty for municipal and state managers in defining and establishing a flow of assistance with hierarchisation of the various levels of treatment.

The limitations related to the integrity and validity of the information in the death notices in the national data bank must be taken into consideration, especially for the death notices for residents of the North and Northeast regions of the country [30]. There are also disparities in the quality of the data between urban and rural areas of Brazilian states as well as in cities of smaller and larger populations [31]. While the quality of death notices and the percentage of under-reporting in Brazil seems to be improving in most states, the results reported for some regions still must be considered with caution, as the proportion of improperly defined causes of death can reach 10% in the Northern and Northeastern states of Brazil, leading to an underestimation of the mortality rates [32, 33]. Finally, the findings must be treated carefully in order to avoid the ecological fallacy of using aggregate results to make any inferences at the individual level.

## Conclusions

This study found differences in the pattern of mortality because of colorectal cancer in large Brazilian cities, where it was observed that the mortality rate was directly proportional to the value of the MHDI and level of urbanisation, and inversely proportional to the inequality indicators, resulting in higher levels in the cities with better socioeconomic conditions.The policy for controlling cancer in Brazil is defined based on the analysis of the morbidity and mortality profiles of the regions. While it is recognised that there are strong differences within the regions i.e. between mesoregions and states, it is necessary to begin by looking at these large areas in order to take initial measures of decentralisation of activities and resources so that regionalisation can be used as an allocation criteria for the installation capacity in the medium term. It is important for one to consider social disparities in order to ensure equity in the management of health policies. The goal should be one of reducing inequities by means of redistribution of the available preventive and diagnostic services in order to reduce the rate of disease caused by colorectal cancer.

## Conflict of interests

The authors declare that there are no conflict of interests, and also no financial support existed in the completion of this study.

## Author contributions

All of the authors participated in the conception and preparation of the article, bibliographic research, data interpretation and analysis, discussion of results, editing, and the final approval of this article.

## Figures and Tables

**Table 1.** Frequency of cities which make up each socioeconomic group by region, Brazil, 2015 (*N* = 287).

Region	*n* (%)		Total (%)	P value^*^
	A (*N* = 235)	B (*N* = 52)		
North	14 (5.9%)	8 (15.5%)	23 (8.0%)	<0.001
Northeast	14 (5.9%)	41 (78.8%)	58 (20.2%)	
Southeast	139 (59.5%)	1 (1.9%)	138 (48.1%)	
South	49 (20.9%)	1 (1.9%)	49 (17.1%)	
West Central	19 (7.8%)	1 (1.9%)	19 (6.6%)	

* Obtained using the exact Fisher test

**Table 2.** Profile of the groups of large cities according to the median and amount of the variance ratio among/within groups (F) of socioeconomic indicators, Brazil, 2015 (*N* = 287).

Socioeconomic indicator	Median for the indicator in the group (DP)		Total median	F
	A	B		
HDI	0.81 (0.026)	0.70 (0.039)	0.78 (0.049)	421.83
Gini coefficient	0.53 (0.053)	0.60 (0.038)	0.56 (0.045)	47.36
Income ratio	13.41 (2.491)	18.99 (2.612)	16.60 (8.937)	235.40
Urbanisation level	92.04 (5.271)	77.65 (5.107)	82.30 (8.974)	251.35
Number of cities	235	52	287^**^	–

** All of the indicators show significant differences (*p* > 0.001)

**Table 3.** Profile of the groups of cities according to median values of the mortality indicators, Brazil, 2015.

Mortality indicator^*^	Median for the indicator in the group (DP^a^)		Total median (DP^a^)	F	P value^c^
	A	B			
TM^b^ because of colorectal cancer	13.90 (4.034)	9.13 (4.657)	12.76 (4.586)	7.49	<0.001

Key:

a DP - pattern deviation

b TM - Mortality rate

c Obtained by means of the Student t test

* Mortality rates adjusted by age range in five year increments and more than 80 years of age.

**Table 4.** Correlations between the socioeconomic indicators and the mortality rate because of colorectal cancer in large Brazilian cities, Brazil, 2015.

Indicators	TM colorectal^a^	HDI^b^	Gini^c^	UrbLevel^d^	Income R^e^
TM colorectal	1	0.383^*^	−0.147^*^	0.372^*^	−0.398^*^
HDI	0.383^*^	1	−0.201^*^	0.547^*^	−0.478^*^
Gini	−0.147^*^	−0.201^*^	1	−0.389^*^	0.356^*^
Urb level	0.372^*^	0.547^*^	−0.389^*^	1	−0.519^*^
Income ratio	−0.398^*^	−0.478^*^	0.356^*^	−0.519^*^	1

Key:

a TM colorectal–mortality rate because of colorectal cancer

b HDI – Human development index

c Gini – Gini coefficient

d Urb Level – Urbanisation level

e Income R – Income ratio

* Statistically significant correlations (*p* < 0.001)
